# Non-Local and Multi-Scale Mechanisms for Image Inpainting

**DOI:** 10.3390/s21093281

**Published:** 2021-05-10

**Authors:** Xu He, Yong Yin

**Affiliations:** School of Microelectronics and Communication Engineering, Chongqing University, Chongqing 400044, China; xuhe8@cqu.edu.cn

**Keywords:** image inpainting, implicit diversified Markov random fields, dense connection of dilated convolution, contextual attention mechanism

## Abstract

Recently, deep learning-based techniques have shown great power in image inpainting especially dealing with squared holes. However, they fail to generate plausible results inside the missing regions for irregular and large holes as there is a lack of understanding between missing regions and existing counterparts. To overcome this limitation, we combine two non-local mechanisms including a contextual attention module (CAM) and an implicit diversified Markov random fields (ID-MRF) loss with a multi-scale architecture which uses several dense fusion blocks (DFB) based on the dense combination of dilated convolution to guide the generative network to restore discontinuous and continuous large masked areas. To prevent color discrepancies and grid-like artifacts, we apply the ID-MRF loss to improve the visual appearance by comparing similarities of long-distance feature patches. To further capture the long-term relationship of different regions in large missing regions, we introduce the CAM. Although CAM has the ability to create plausible results via reconstructing refined features, it depends on initial predicted results. Hence, we employ the DFB to obtain larger and more effective receptive fields, which benefits to predict more precise and fine-grained information for CAM. Extensive experiments on two widely-used datasets demonstrate that our proposed framework significantly outperforms the state-of-the-art approaches both in quantity and quality.

## 1. Introduction

Image inpainting, which synthesizes semantically reasonable and visually plausible contents in the damaged regions from existing areas, has attracted great attention in recent years. High-quality image inpainting can be capable of benefiting a wild range of applications such as unwanted-object-removal [1,2] and photos restoring [3]. Not only it is necessary to reconstruct textures and contents, but it is also crucial to have insight into the scene and objects that will be completed. Despite of many years research, image inpainting is still a challenging task in the field of computer vision as there is an inverse ill-posed problem [4] in this technique. 

Generally, image inpainting approaches can be classified into three categories, diffusion-based method [5], patch-based method [6] and deep-learning-based method [7]. The first two methods depend on spreading and copying known information, hence they have an inferior ability to acquire high-level semantic features. Recently, deep-learning-based approaches such as convolutional neural networks (CNN) [8] and generative adversarial networks (GAN) [9] have exhausted the powerful capability of reconstructing target regions from surrounding areas. 

Pathak et al. [10] designed a model based on the CNN termed as context encoder, which consists of an encoder to capture the context of an image into a compact latent feature representation and a decoder which utilizes that representation to predict the target region. Although it can achieve promising results, some exquisite details are ignored due to the more attention on recovering structure information rather than fine details. To tackle this issue, many methods adopted a two-stage architecture network. For instance, Yu et al. [11] introduced a coarse-to-fine framework to rough out the missing contents and a refined complete module to capture high-level features from known areas. Based on this network, Iizuka et al. [12] designed a global discriminator and a local discriminator to distinguish between real images and repaired images respectively, which can maintain the coherence of missing areas and surrounding regions. Above-mentioned methods mainly focus on rectangular areas and assume that the missing area is in the middle of the image. However, this hypothesis is limited to research and cannot be widely used in practice. Recently, massive methods have been investigated to deal with this problem. For example, Liu et al. [13] first put forward the mechanism of partial convolution (PConv) which incorporates re-normalized convolution and a mask-update operation to replace convolutional layers. Yu et al. [14] presented a gated convolution for free-form image completion. Furthermore, Ma et al. [15] designed a region-wise network to boost the capability of the generative model to adaptively learn feature presentations in different regions. Those methods have achieved promising performance on small proportion corrupted regions, while show insufficiency when the incomplete regions occupy a large proportion. Hence, they will inevitably lead to artifacts such as color discrepancies and blurriness.

To overcome the above-mentioned problems, we mainly focus on arbitrary and large image defects. Our work builds upon the recently proposed region-wise approach [15], which employed a region-wise convolution mechanism and trained the network with a joint loss that consists of reconstruction losses, a style loss, a correlation loss and an adversarial loss. It performs well when the missing region is discontinuous, while suffers from obvious grid-shaped artifacts in larger and continuous occluded image regions. This phenomenon is probably caused by the correlation loss and the style loss since they all use the gram matrix which pays attention to capturing pixel-wise correlations rather than taking the global consistency into account. Furthermore, they proposed an adversarial network to mitigate large area artifacts. However, there is no obvious improvement for visual performance. Another limitation is that lots of results containing over-smooth and incomplete structures will be obtained when the damaged area is large. We speculate that the original model does not have enough capacity to learn feature representations of different regions. Based on those observations, our proposed approach addresses those points and further achieves desirable results by focusing on non-local relationships and multi-scale information. More specifically, we retain original region-wise convolutions and the two-stage structure in paper [15] for the following reasons. Region-wise convolution can perform different operations for different regions. However, only adopted region-wise convolution framework will obtain blurry results. Hence, it is indispensable to incorporate the refinement network to infer more precise details. Based on the above-mentioned structure, we first utilized the pretrained VGG model combining with implicit diversified Markov random fields to substitute Gram-matrix-matching, which can alleviate the effects of grid-like shape artifacts. However, the repaired results will be blurred when the missing regions occupy a large proportion. Hence, we introduced the contextual attention module (CAM) to capture features in background patches and propagate the spatial coherency of attention. However, we find that the results will contain incorrect textures and incomplete structures. We analyze that the original cascade dilated convolution cannot provide abundant and accurate information for the CAM. To tackle this problem, we introduced several dense fusion blocks (DFB) to replace the original cascade dilated convolution and extract multi-scale features for CAM simultaneously. In conclusion, the combination of CAM and DFB can achieve the visual authenticity and perceptual plausibility of results. 

We evaluate and analyze our proposed method on two standard datasets including CelebA-HQ and Paris StreetView. Meanwhile, we compare our model with the state-of-art schemes and provide experimental results to verify the effectiveness of our proposed method. 

The main contributions of this paper are summarized as follows:

- We aim to solve image inpainting tasks for randomly missing regions with a large proportion and employ an ID-MRF loss to tackle grid-shape artifacts and color discrepancy caused by style loss and correlation loss.
- We innovatively combine the CAM with the DFB module to assist our network to generate precise and fine-grained contents by borrowing features from distant spatial location and extracting multi-scale features.
- Experiments on multiple benchmark datasets intuitively show that our method is able to achieve competitive results.

## 2. Related Work

Recently, a great deal of literature has proposed numerous methods for image inpainting. In this section, we will mainly introduce a few methods related to non-local and multi-scale mechanisms. 

### 2.1. Non-Local Mechanisms

For discontinuous missing regions, semantic information can be easily inferred from the background. However, it is a challenging task to repair large and continuous masked areas due to the huge gap between empty missing regions and corresponding possible recovered contents. Attention mechanism based on the relationship between contextual and missing regions has been often used in the task of image inpainting. For instance, Yu et al. [11] proposed a coarse-to-fine generative adversarial network and appended a contextual attention module to learn feature presentations via matching patches from background information. However, once the wrong information is captured in the first stage, it will cause the propagation of the error. On this basis, Sagong et al. [16] proposed a parallel extended-decoder path with a modified contextual attention module to reduce the number of convolution operations and create a higher-quality inpainting result simultaneously. For capturing long-range spatial dependencies, the self-attention mechanism [17] based on the non-local network [18] was wildly adopted. For instance, Uddin et al. [19] designed a global and a local attention architecture to obtain global and local coherent information. Yang et al. [20] exploited a self-attention mechanism and integrated it with structural information. Liu et al. [21] proposed a non-local module to capture a deeper relationship of different regions by using a self-attention framework. However, the non-local module was originally designed for the task of classification and this operation is not sufficient to significantly improve the performance of our framework. In addition to using the attention mechanism to obtain non-local information, a pre-trained model of the VGG network [22] has been wildly adopted to extract non-local features by calculating style loss. The essence of style loss is learning the relationship of existed and unknown regions by using the Gram-matrix to calculate the pixel relevance. Although it can preserve the high-frequency details, there is a tendency to produce grid-shape artifacts and contents that is inconsistent with the background. With the purpose of further successfully generating high-quality images, several studies concentrated on taking advantage of similarities of patches as loss function to learn non-local features. For example, Yang et al. [23] proposed a style transfer based on an MRF method to promote feature fusion, structure completion and texture reconstruction. In particular, Wang et al. [24] proposed an MRF-based non-local loss to encourage network to produce high-quality results by considering content consistency and texture similarity. 

### 2.2. Multi-Scale Mechanisms

Currently, multi-scale-based methods have shown a significant development of applications in image inpainting. For instance, Wang et al. [25] introduced a Laplacian-pyramid model to progressively restore images with different resolutions. Mo et al. [26] introduced several multi-scale discriminators to generate the results containing more multi-scale information. Wang et al. [24] proposed a multi-column convolutional neural network to enlarge the receptive fields by applying different convolutional kernel size. However, those methods will be likely to cause resource-consuming and suffer from additional parameters. The common operation of aggregating multi-scale information and reducing resource consumption is to enlarge receptive fields by using dilated convolution. In the work of [12], they replaced the original channel-wise fully connected layer by a cascaded dilated convolution to broaden the receptive field. To further learn multi-scale features, Hui et al. [27] utilized dense combinations of dilated convolutions and different dilated rates to learn larger and more effective receptive fields, which is vital to infer reasonable structures and contents.

## 3. Proposed Methods

**Figure 1:**
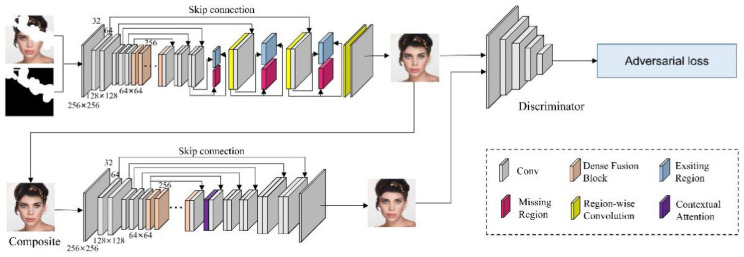
The overall architecture of our method. Region-wise convolution indicates using different convolution filters for different regions, more details can be found in paper [15]. In this architecture, 256 by 256 and 32 denote the size and channels of the feature map respectively.

In this section, we firstly described the process of our method based on the model of generative adversarial network. Then we introduced the details of the contextual attention mechanism and the dense connection architecture of dilated convolution. Finally, objective loss functions including the reconstruction loss, the ID-MRF loss and the adversarial loss are presented in detail. An overall framework of our method is displayed in Figure 1.

### 3.1. The Architecture of Our Framework

As depicted in Figure 1, we take the architecture proposed by Ma et al. [15] as the backbone of our generator which is composed of several region-wise convolutions and the cascaded dilated convolution based on the coarse-to-fine structure. On this basis, we introduce the contextual attention module (CAM) and use the dense connection of dilated convolution as dense fusion block (DFB) to replace the original cascaded convolution. The CAM is not suitable for the coarse stage as this phase cannot provide enough accurate and delicate information for the CAM to borrow and propagate. Moreover, ordinary cascaded dilated convolution cannot extract multi-scale features of the image. Inspired by those observations, we integrate DFB into coarse and refinement stages and only employ the CAM in the refinement stage. In addition, we only embed one CAM as it is sufficient for feature borrowing and reconstruction. Generally, the combination of DFB and CAM can synthesize more fine-grained and better results. Moreover, in the work of [11] and [16], the attention module is used in one branch of the parallel network. However, we find it is not suitable for our architecture since our model has a strong dependence on skip connections and dilated convolutions. Based on this observation, we design a novel refinement framework to improve the robustness of the inpainting model and synthesize realistic contents simultaneously. It is worth emphasizing that the input of CAM is the concatenation of the convolutional layers before and after the dilated convolution. As shown in Figure 1, given a ground truth image $I_{gt}$ and a binary mask M which denotes damaged areas (0 for missing regions, 1 for existing counterparts). The corrupted image is

(1) $$\bar{I_{gt}}=I_{gt}⊙M$$

where the symbol ⊙ denotes the multiplication of corresponding elements of two matrices. We feed the concatenation of $\bar{I_{gt}}$ and M as inputs to the coarse network instead of$\;\bar{I_{gt}}$, which is beneficial for the network to concentrate more on valid pixels. Then the predicted image $I_{pred}^{1}$ as the same resolution as the original image will be obtained. We integrate the masked area in $I_{pred}^{1}$ with the opposite regions in the background as the composite image of the first stage. It can be denoted by the equation as below:

(2) $$I_{comp}^{1}=\bar{I_{gt}}+I_{pred}^{1}⊙\left(1-M\right)$$

Then the $I_{comp}^{1}$ is sent to the refinement network to provide more information and the model can yield the refined image $I_{pred}^{2}$, which is used to obtain

(3) $$I_{comp}^{2}=\bar{I_{gt}}+I_{pred}^{2}⊙\left(1-M\right)$$

Moreover, we only consider the local predicted region in the phase of the adversary. Specifically, two local predicted images $I_{pred}^{1}⊙\left(1-M\right)$, $I_{pred}^{2}⊙\left(1-M\right)$ in every stage are feed together into the discriminator to enhance the capability of generator. In addition, we adopt the recently proposed technology of spectral normalization [28] which controls the Lipschitz constant of the discriminator.

### 3.2. Contextual Attention Mechanism 

Recently, the attention mechanism has been widely used in image inpainting tasks and exhibits a great potential in the generation of high-quality images. Since there is a strong necessary to apply a non-local mechanism to deal with continuous and large missing regions, we employ a contextual attention mechanism (CAM) in the refinement network to enhance the power of the generator to obtain sharper and pleasing results from initial prediction models. Yu et al. [11] firstly proposed the contextual attention module which borrows the patches from the background to fill holes. However, it utilizes the cosine similarity to match similar patches, which may influence the feature extraction due to the normalization operation. Furthermore, Sagong et al. [16] modified this module by replacing the cosine similarity with Euclidean distance. It is more feasible to match and propagate more reasonable contents since the Euclidean distance can not only take the angle of different patches into account, but also consider the size of them. We refer to this method to achieve the propagation of non-local features. The process of the attention mechanism can be defined as follows:

The first step is to divide the feature maps into background and foreground regions: background indicates known regions and foreground denotes opposite counterparts. We can obtain the background area though multiplying the mask by feature maps. Then we extract patches from different regions and reshape those patches of background as convolutional filters. Then we measure the similarity score ${\bar{d}}_{\left(x,y\right),\left(\;x\prime ,\;y\prime \right)}$ between foreground patch $\left(f_{x,y}\right)$ and background patch $\left(b_{\;x\prime ,\;y\prime }\right)$ by the function:

(4) $${\bar{d}}_{\left(x,y\right),\left(x^{\prime },y^{\prime }\right)}=tanh(-\left(\frac{d_{\left(x,y\right),\left(x^{\prime },y^{\prime }\right)}-m\left(d_{\left(x,y\right),\left(x^{\prime },y^{\prime }\right)}\right)}{\sigma \left(d_{\left(x,y\right),\left(x^{\prime },y^{\prime }\right)}\right)}\right))$$

where

(5) $$d_{\left(x,y\right),\left(x^{\prime },y^{\prime }\right)}=||f_{x,y}-b_{x^{\prime },y^{\prime }}||$$

where m and σ indicate the constant value.

Finally, the foreground region is reconstructed from the weighted sum of background patches, and the importance of the background patch is judged by the similarity score. With the assistance of the tanh function, our model has the ability to accurately distinguish background and foreground, so as to better match and propagate features. Moreover, this module plays an important role in alleviating the influence of redundant information and synthesizing the satisfactory results by adaptively differentiating and fusing long-range spatial information.

### 3.3. Dense Connection of Dilated Convolution

**Figure 2:**
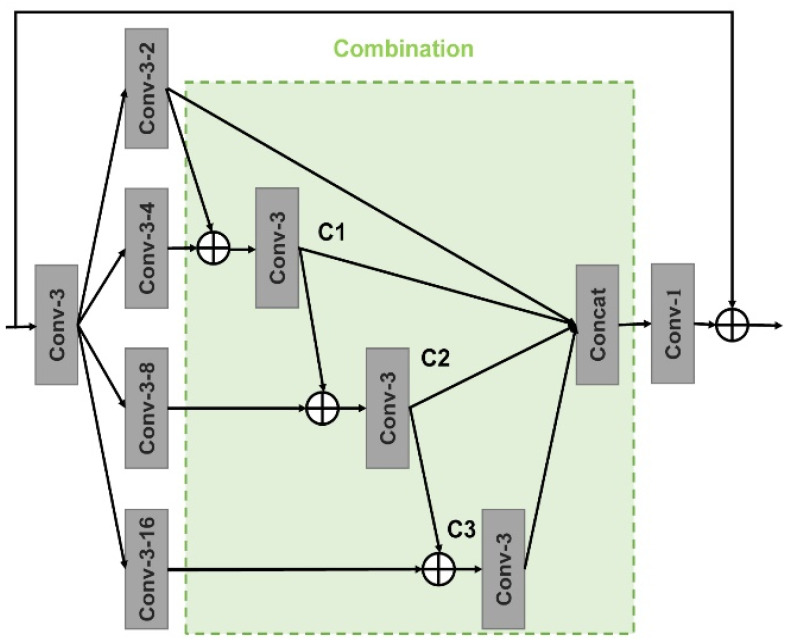
The framework of the dense fusion block. “Conv-3-2” indicates the 3 by 3 convolution layer and the dilated rate is 2. ⊕ is element-wise summation. The output channels of all convolutional layers are 64, except for the last layer which is 256.

Although the CAM has delivered a remarkable improvement performance in the reconstruction of structures and contents, it depends on the accuracy of initially predicted images. In addition, it has been proved that if the coarse network performs not well, the refinement phase will take advantage of irrelevant information and feature patches to match and attend [16]. We also find that the skip connection in our framework benefits the network to learn more valid and deeper information. Inspired by those observations, we design a dense connection of dilated convolution which is similar to the structure in paper [27]. As illustrated in Figure 1, the middle layers of the network consist of a series of dense fusion blocks (DFB) based on the dense connection of dilated convolutions and the concrete structure of every DFB is presented in Figure 2. Different from the common cascaded dilated convolutions [11] using various dilated rates, which may restrict the range flexibility of the generator, our dense combination has a large respective field and can adaptively learn more effective information. Specifically, a 3 by 3 convolution is employed to reduce parameters of input feature maps and concentrate on more valid features by decreasing the channels to a quarter of the original counterparts. Then there are four dilated convolution branches with different dilated rates of 2, 4, 8 and 16, respectively, and every convolution utilizes 3 by 3 kernel size. Suppose $x_{i}\left\{i=1,2,3,4\right\}$ indicates the four branches, C(⋅) indicates the convolutional operator and $y_{i}\left\{i=1,2,3,4\right\}$ denotes the output after C(⋅). The process of our dense connection can be demonstrated as follows:

(6) $$y_{i}=\{\begin{array}{ll} x_{i} & i=1\\ C_{i}\left(x_{i-1}+x_{i}\right) & i=2\\ C_{i}\left(y_{i-1}+x_{i}\right) & 2<i\leq 4 \end{array}$$

We can obtain multi-scale information by connecting all the outputs $y_{1},y_{2},y_{3},y_{4}$. Then a 1 by 1 convolution is adopted to aggregate features. It is worth notable that all the convolution layers in the DFB have the same structure as the other counterparts in our architecture. A series of DFBs enjoy the ability to preserve multi-scale information and increase the richness of extracted features by enlarging the receptive fields.

### 3.4. Loss Functions

The task of image inpainting is an ill-posed problem in that there are a number of possible results. Therefore, it is crucial to use loss functions to select the most reasonable and realistic one. In our experiment, we rely on several loss functions to optimize networks in the training process.

#### 3.4.1. Reconstruction Loss

Reconstruction loss is a straightforward method that can measure pixel-wise differences between the predicted region and the associated surroundings. We prefer to adopt $L_{1}$ distance to calculate the reconstruction loss rather than $L_{2}$ distance as the latter will produce images with much blurriness [29]. We will verify this conclusion in the ablation studies. In our two-stage model, the overall reconstruction losses can be expressed as follows:

(7) $$L_{re}=||I_{pred}^{1}-I_{gt}||{}_{1}+||I_{pred}^{2}-I_{gt}||{}_{1}$$

The pixel reconstruction loss can guarantee the consistency of contents in the generated area and the background area. Moreover, this function can benefit to reconstruct initial structural information while some high-frequency details will be ignored.

#### 3.4.2. ID-MRF Loss

The style loss and correlation loss in paper [15] are prone to cause grid-shape artifacts and color discrepancy when repairing large continuous masked regions. We also find that the repaired target region will be very blurry if removing loss functions which focus on the relationship of different parts. Aiming to address those problems and motivated by the work of paper [30] and [24], we adopted an ID-MRF loss to capture complex image layouts and provide plausible contents with the same pattern in visual and style to the ground truth.

Suppose $I_{pred}^{1}$ and $I_{gt}$ be the predicted image and the ground-truth image respectively, $\Phi_{p}^{l}$ is the feature maps derived from the $l_{th}$ feature layer of a pretrained VGG model. Similarly, $\Phi_{GT}^{l}$ is the feature maps of the original image. Let m and n indicate one of the patches extracted from $\Phi_{p}^{l}$ and $\Phi_{GT}^{l}$, respectively. The relative similarity between m and n is defined as

(8) $$RS(m,n)=\exp((\frac{\mu (m,n)}{\underset{\nu \in R_{n}(\Phi_{GT}^{l})}{\max}\mu (m,\nu )+\delta })/s)$$

where μ(⋅,⋅) indicates the cosine similarity. $R_{n}\left(\Phi_{GT}^{l}\right)$ denotes all the neural patches in $\Phi_{GT}^{l}$ excepting for n. δ and s indicate positive constants. Then we normalize RS(m,n) to

(9) $$\bar{RS}\left(m,n\right)=RS\left(m,n\right)/\underset{v\in R_{n}\left(\Phi_{GT}^{l}\right)}{\sum }RS\left(m,v\right)$$

The ID-MRF loss of $l_{th}$ feature layer between $\Phi_{p}^{l}$ and $\Phi_{GT}^{l}$ is defined as

(10) $$L_{M}\left(l\right)=-log(\frac{1}{h}\underset{n\in \Phi_{GT}^{l}}{\sum }\underset{m\in \Phi_{P}^{l}}{max}\bar{RS}\left(m,n\right))$$

where h is normalization constant. Different from common cosine similarity, the ID-MRF loss concentrates on relative distance which benefits to find high-quantity patches in the neighborhood. By minimizing $L_{M}\left(l\right)$, the process of m in $\Phi_{p}^{l}$ seeking for some non-local similar candidates in $\Phi_{GT}^{l}$ will constrain the network to generate images closer to the real counterparts.

Let the predicted image is $I_{pred}^{2}$. Then it is projected to a more advanced feature space using pre-trained VGG16 on ImageNet. We use conv3_2 and conv4_2 to indicate image texture and conv4_2 to describe semantic structures. The ID-MRF loss is defined as:

(11) $$L_{mrf}=L_{M}\left(conv4\_2\right)+\overset{4}{\underset{i=3}{\sum }}L_{M}\left(convi\_2\right)$$

Since the correlation loss and style loss are pixel-wise methods rather than patch-wise, our ID-MRF loss has the ability to establish the relationship of long-term contents.

#### 3.4.3. Adversarial Loss

Only relying on generator cannot guarantee to yield plausible results. The experiments in [31] have confirmed that the adversarial network can benefit to remove grid-like artifacts. Motivated by this research and aiming to produce pleasing results, we adopted a discriminator to encourage the generator to synthesize visually consistent results. Given $I_{gt}$, $I_{pred}^{1}$,and $I_{pred}^{2}$, we penalize the predicted missing regions rather than the entire image and concatenate those areas with the corresponding mask as inputs of the discriminator. To sum up, the learning objective for the discriminator in our experiments is formulated as:

(12) $$\begin{array}{cc} L_{ad\dot{v}} & =\alpha E\left(D\left(I_{gt}⊙\left(1-M\right),M\right)\right)\\ & +E\left(1-D\left(I_{pred}^{1}⊙\left(1-M\right),M\right)\right)\\ & +E\left(1-D\left(I_{pred}^{2}⊙\left(1-M\right),M\right)\right) \end{array}$$

where D and M indicate discriminator and the mask respectively. We set α as 0.01.

For the generator, it struggles to improve the quality of synthesis results to fool the discriminator, while the task of the discriminator is to judge whether the predicted image is true or fake until the discriminator is indistinguishable from those images obtained by the generator.

#### 3.4.4. Overall Loss

Finally, we obtain the hybrid loss function which is a linear combination of the construction loss $L_{re}$, the ID-MRF loss $L_{mrf}$ and the adversarial loss $L_{adv}$.

(13) $$L=\lambda_{1}L_{re}+\lambda_{2}L_{mrf}+\lambda_{3}L_{adv}$$

where $\lambda_{1}$, $\lambda_{2}$, $\lambda_{3}$ are weights of different loss components. Our joint loss function can ensure the generator to produce semantically-reasonable and visually-realistic results.

## 4. Experiments

In this section, we present the datasets used in this work and our experimental implementation. We also compare our approach with several state-of-the-art image inpainting methods to evaluate the effectiveness of our model qualitatively and quantitatively. Finally, we conduct ablation studies to examine the effect of different components in our model.

### 4.1. Datasets and Masks

We validate our method on two public and widely-used datasets: CelebA-HQ dataset and Paris StreetView dataset. The former focuses on human face and contains 30,000 images, the latter collected from street views of Paris contains 14,900 training images and 100 test images. We use the original train, test and validation splits for these two datasets. Moreover, we adopt an algorithm to generate irregular masks during training and testing, which is more suitable for the situation of natural damaged images and can avoid over-fitting.

### 4.2. Implementation Details

Our proposed framework is implemented in Tensorflow. The size of input images is 256 by 256 and the batch size is 4. Our model is optimized by the Adam algorithm with a learning rate of $1\times 10^{-4}$ and $\beta_{1}=0.5$, $\beta_{2}=0.9$. We train our model on the NVIDIA 2070 GPU (8GB) and NVIDIA 2080Ti GPU (11GB). To stable the process of training, we divide it into two steps. Specifically, we train our model without the adversarial loss for the former 20 epochs and append it for the latter 10 epochs. Since we find that the larger proportion of our MRF loss will cause the propagation of incorrect contents, while over-smooth and blurry results will be obtained when their proportion is lower. Motivated by this observation, the trade-off parameters $\lambda_{1}$, $\lambda_{2}$ and $\lambda_{3}$ are set to 20, 1 and 0 in the first step. In the second step of training, we deploy the adversarial network and set $\lambda_{1}$ as 10, $\lambda_{2}$ as 1, $\lambda_{3}$ as 1. The masked image includes missing regions with variable numbers, sizes, shapes and locations during every iteration. In particular, the proportion of arbitrary damaged areas varies from 0% to 40% during training. In addition, we experimentally set the number of DFB to 8. It takes 2 s to predict missing regions with any shapes and 1 day to train 20 epochs of 28,000 high-resolution images.

### 4.3. Comparative Experiments

We apply our network to perform qualitative and quantitative comparison experiments with several state-of-the-art methods including contextual attention (CA) [11], partial convolution (PConv) [13], GatedConv (GC) [14], EdgeConnect (EC) [32], Pluralistic Image Completion (PIC) [33] and Region-Wise Conv (RWC) [15]. To fairly evaluate, we carry out the experiments on discontinuous and continuous missing regions and every test image has the corresponding mask. For CA, GC and RWC, we train their models on CelebA-HQ dataset and Paris StreetView dataset with the released code. For EC and PIC, we directly adopt the pre-trained model as our training results perform not as good as the released results. As to PConv, we refer to the implementation on github (https://github.com/MathiasGruber/PConv-Keras, accessed on 15 December 2020) and follow the suggestions of authors for training since there are no public codes.

#### 4.3.1. Qualitative Comparison

**Figure 3:**
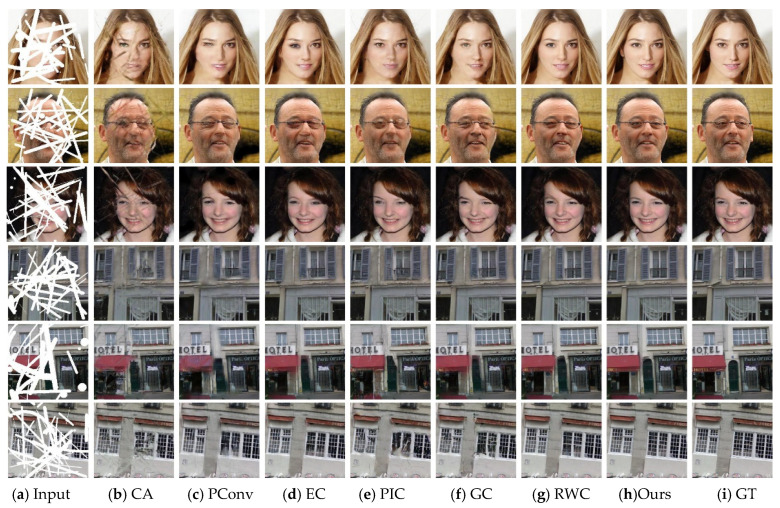
Qualitative comparisons of different methods on discontinuous missing areas.

**Figure 4:**
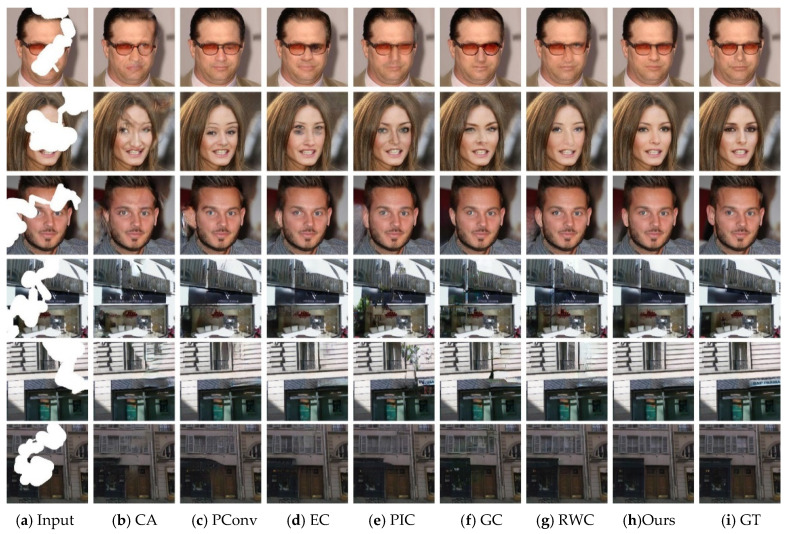
Qualitative comparisons of different methods on continuous missing areas.

Figure 3 and Figure 4 present the inpainting results of different state-of-the-art approaches on some samples selected from CelebA-HQ and Paris StreetView datasets. GT indicates the ground-truth image. To make a comprehensive and objective comparison of various methods, we explore the effect of different missing ratios of masks ranging from 0–40%, which is consistent with the training phase. Observing from Figure 3 and Figure 4, inpainting results of CA suffer from visible distortions and inconsistency because it is originally designed for restoring regular missing holes. Among the rest of algorithms, EC reconstructs images with more accurate and intact structures when the missing region is narrow, but it still faces artifacts compared to the ground truth. PIC is proposed for the task of generating reasonable and diverse results hence it is difficult to approximate the true distribution of images. Although PConv and GC are designed to deal with irregular missing regions, they fail to repair some structure information such as the eye and some details in building. RWC is designed to cope with arbitrary masked images. It can successfully repair correct contents when the missing region is discontinuous, while brings strong grid-like artifacts and incomplete structures in large continuous missing parts. For face images, our model has the ability to repair missing glasses, synthesize symmetrical eyes and predicted vivid results. For Paris StreetView datasets, our method exhibits a superior performance with more intact structures and exquisite details. From what has been discussed above, we can draw the conclusion that our model exhibits competitive results with fine-grained details, pleasing textures and consistent structures with the help of the combination of the ID-MRF loss, dense fusion blocks and the contextual attention module. 

#### 4.3.2. Quantitative Comparison

We evaluate our model with five commonly used image quality metrics: the L1 loss, the L2 loss, the peak signal-to-noise ratio (PSNR), the structure similarity index (SSIM) and the Frechet Inception Distance (FID). Specifically, the PSNR measures the difference in pixel values of two images, and SSIM measures the similarities between the reconstructed image and the original image. Larger PSNR and SSIM values indicate smaller gaps between the generated image and the ground truth. Moreover, the L1 loss can reflect the ability to create the image as possible as closer to the real counterpart. L2 indicates the mean square error. The value of FID was introduced to calculate the Wasserstein-2 distance between the two distributions by utilizing the pre-trained Inception-V3 model [34]. 

Table 1 and Table 2 list full comparisons of all discussed methods in terms of five metrics with different ratios of irregular masks. As is illustrated in those Tables, we can obtain the conclusion that it is more difficult to repair the missing regions on Paris StreetView dataset since the values of PSNR and SSIM on it are commonly lower than the counterparts on CelebA-HQ dataset and the scores of L1 loss, L2 loss and FID are higher. For discontinuous damaged regions, it is quite obvious that our method shows a relative improvement in terms of all indicators. Moreover, CA shows a competitive value of PSNR at the mask ratio of 0–10%. At the same time, it faces a strong performance degradation accompanying with the increasing damaged regions. PConv, ED and PIC show the almost same performance on those two datasets and gain inferior indicators compared with GC, RWC and ours since they lake the deep understanding of semantic information and the correlation between existing regions and surroundings. The quantitative results demonstrate that our model achieves better scores in most cases. It is worth noting that the average value of PSNR increased by 1.28 for continuous missing regions on Paris StreetView dataset.

### 4.4. Ablation Studies

In this section, we analyze the contribution of each component in our proposed model to the final performance by presenting the inpainting results quantitatively and qualitatively. First, we respectively compare the repair results under the constraints of L1 and L2 reconstruction loss. Then we present the results obtained by the correlation loss (CL), the style loss (SL), the ID-MRF loss (IM), the combination of IM and adversarial loss (IM+AD). Subsequently, we utilize IM+AD as baseline (BL) and append the attention module into BL (BL+AT) to validate its effectiveness. Finally, we replace the cascaded dilated convolution in BL with several dense fusion blocks (DFB) to identify its role in our whole model, which can be expressed as BL(Rcdc)+DFB.

**Figure 5:**
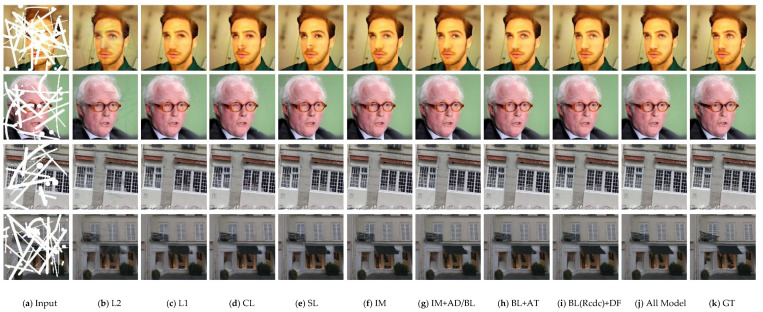
Qualitative results of ablation studies on discontinuous missing regions. (Best viewed with zoom-in.)

**Figure 6:**
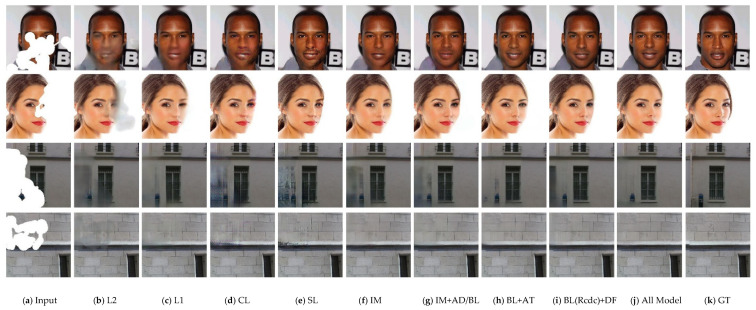
Qualitative results of ablation studies on continuous missing regions. (Best viewed with zoom-in.)

It can be seen from Figure 5 and Figure 6 that the L2 reconstructed loss suffers from more severe blurring and shadow-like artifacts than L1 loss. Moreover, the CL will incur obvious color discrepancy and SL tends to produce images containing more high-resolution details that are inconsistent with non-missing regions such as grid-like and aliasing artifacts. The reason for this phenomenon may be that those losses lack the advanced semantic feature and the reasonable extraction of spatial information to guide image synthesis. By utilizing the ID-MRF loss, the grid-like artifacts can be alleviated while over-smooth and blurry results will be obtained in large missing regions. Adversarial loss can further mitigate the effect of blurriness, but it is far from meeting the visual requirements. Moreover, pleasing contents and reasonable structures cannot be guaranteed by using either AT or DFB. By integrating them, the repairing images show an obvious visual enhancement on pleasing structures and textures. 

As shown in Table 3 and Table 4, the value of L2 loss is far worse than the L1 loss in terms of those five indicators, which is consistent with visual appearance. Compared with the correlation loss and style loss, most of the metrics are enhanced by a large margin under the ID-MRF loss constraint. This phenomenon indicates that the loss based on the non-local mechanism is more appropriate for improving the high relevance between hole and background regions than those counterparts based on pixel-wise correlations. By gradually embedding adversarial loss and the attention module and using the BL(Rcdc)+DFB module, the performance of our model is improved stably. In particular, the combination of CAM and DFB can achieve a superior performance than using any of them alone. We attribute this phenomenon to the joint effect of the enlargement of the receptive field and the reconstruction of non-local relevant features. 

## 5. Conclusions and Future Work

This paper combines two non-local mechanisms which consist of the ID-MRF loss and the contextual attention module (CAM) with a multi-scale method named the dense fusion block (DFB) which relies on the dense connection of dilated convolution. Under the interaction of the various proposed mechanisms, our model can repair large continuous and discontinuous missing regions at the same time. Specifically, the ID-MRF loss can suppress color discrepancies and grid-like artifacts cause by the correlation loss and the style loss in the task of image inpainting. On this basis, we integrate the CAM with DFB to further predict high-quality results with more fine details. The former can capture long-term spatial information by borrowing or copying the feature information from known background patches and the latter can extract multi-scale features by enlarging receptive fields. Experimental results demonstrate that our proposed model can achieve superior performance than state-of-the-art methods both in quantity and quality.

Although DFB have an impact on the performance improvement of our model, this contribution is insufficient, and they require considerable computational resources due to the structure of dense connection. Hence, further improvements can be achieved by reducing the parameters of the network. In addition, the receptive-field-aware module [35] demonstrates a strong capability of enlarging the receptive field in the task of image segmentation. It will be a very meaningful work to combine this technology with our model. Thus, future research will focus on how to apply the receptive-field-aware framework to image inpainting.

## Figures and Tables

**Table 1:** Quantitative comparisons on discontinues missing region, where the **bold** indicates the best performance, and the underline denotes the sub-optimal results, + indicates the higher is better, while—indicates the lower is better.

	MASK	CelebA-HQ							Paris StreetView						
		CA	PConv	EC	PIC	GC	RWC	Ours	CA	PConv	EC	PIC	GC	RWC	Ours
PSNR ^+^	0–10%	34.89	34.24	34.58	34.69	39.28	41.76	**42.25**	35.30	34.20	34.56	34.02	37.85	41.52	**42.19**
	10–20%	27.54	31.01	31.22	31.31	32.65	34.32	**34.95**	28.83	30.52	30.91	30.26	30.97	34.26	**35.11**
	20–30%	24.14	28.21	28.58	28.61	29.07	30.46	**31.18**	25.56	27.67	28.13	27.30	27.01	30.15	**31.12**
	30–40%	22.83	26.95	27.35	27.56	27.92	29.45	**30.18**	23.85	26.31	26.70	25.83	25.96	29.08	**30.04**
SSIM ^+^	0–10%	0.972	0.938	0.945	0.944	0.984	0.990	**0.991**	0.972	0.948	0.950	0.947	0.983	0.989	**0.990**
	10–20%	0.904	0.908	0.917	0.912	0.950	0.964	**0.966**	0.902	0.909	0.915	0.904	0.937	0.961	**0.965**
	20–30%	0.838	0.872	0.879	0.876	0.908	0.930	**0.935**	0.835	0.869	0.871	0.850	0.873	0.918	**0.927**
	30–40%	0.763	0.839	0.847	0.843	0.873	0.905	**0.913**	0.754	0.810	0.829	0.799	0.828	0.892	**0.904**
L1^−^(10^−3^)	0–10%	5.40	13.40	12.94	13.00	4.10	1.63	**1.56**	5.06	13.34	13.19	13.48	4.26	2.50	**1.61**
	10–20%	18.09	16.22	16.00	6.03	8.68	5.67	**5.37**	12.49	17.13	16.79	17.56	9.92	6.42	**5.24**
	20–30%	24.43	21.03	20.33	20.18	16.82	10.30	**9.63**	21.46	22.17	21.78	23.64	18.20	12.11	**10.34**
	30–40%	32.98	24.12	23.29	23.08	18.40	13.59	**12.67**	29.93	27.09	25.99	28.37	23.22	15.47	**13.43**
L2^−^(10^−3^)	0–10%	0.61	0.54	0.50	0.48	0.19	0.13	**0.11**	0.42	0.55	0.52	0.58	0.29	0.16	**0.13**
	10–20%	2.25	1.12	0.98	0.95	0.69	0.49	**0.42**	1.51	1.19	1.13	1.27	1.09	0.59	**0.49**
	20–30%	4.64	1.94	1.75	1.70	1.58	1.13	**0.96**	3.28	2.33	2.10	2.54	2.73	1.45	**1.18**
	30–40%	6.19	2.55	2.23	2.11	1.95	1.39	**1.17**	4.68	3.15	2.80	3.38	3.37	1.83	**1.49**
FID^−^	0–10%	1.97	1.58	1.47	1.37	0.37	0.23	**0.18**	8.32	5.52	4.47	6.12	3.06	1.55	**1.13**
	10–20%	8.95	2.77	2.61	2.29	1.37	0.84	**0.70**	25.37	13.12	10.33	14.06	12.66	6.24	**4.58**
	20–30%	13.98	4.26	3.74	3.20	2.40	1.62	**1.38**	36.14	17.53	15.67	21.94	24.58	13.68	**9.90**
	30–40%	27.06	6.18	5.40	4.36	3.55	2.14	**1.82**	57.93	30.06	26.26	34.20	34.18	17.00	**12.98**

**Table 2:** Quantitative comparisons on continues missing region, where the **bold** indicates the best performance, and the underline denotes the sub-optimal results, + indicates the higher is better, while—indicates the lower is better.

	MASK	CelebA-HQ							Paris Street View						
		CA	PConv	EC	PIC	GC	RWC	Ours	CA	PConv	EC	PIC	GC	RWC	Ours
PSNR ^+^	0–10%	32.11	32.02	32.70	32.91	36.43	37.70	**38.30**	32.83	31.48	33.09	32.30	33.98	36.18	**37.69**
	10–20%	25.33	27.33	28.05	28.00	29.00	29.54	**30.02**	26.39	27.14	28.74	27.18	27.28	29.32	**30.58**
	20–30%	22.86	24.59	25.11	24.81	25.63	26.10	**26.61**	23.40	24.47	26.02	24.26	24.33	26.13	**27.50**
	30–40%	20.21	21.32	21.99	21.58	22.31	22.80	**23.39**	20.69	21.73	23.24	21.60	21.79	23.24	**24.31**
SSIM ^+^	0–10%	0.971	0.936	0.945	0.942	0.979	**0.982**	**0.982**	0.962	0.901	0.933	0.926	0.960	0.977	**0.981**
	10–20%	0.905	0.898	0.906	0.901	0.944	0.944	**0.947**	0.900	0.880	0.895	0.881	0.903	0.928	**0.940**
	20–30%	0.863	0.845	0.866	0.853	0.896	0.897	**0.903**	0.837	0.833	0.844	0.828	0.847	0.872	**0.891**
	30–40%	0.790	0.790	0.801	0.792	0.835	0.837	**0.849**	0.756	0.757	0.782	0.748	0.770	0.808	**0.836**
L1^−^(10^−3^)	0–10%	7.15	15.38	14.26	14.21	4.95	2.84	**2.65**	6.28	15.10	14.14	14.83	5.78	4.15	**2.83**
	10–20%	17.26	20.25	19.77	19.91	11.05	8.85	**8.26**	15.60	22.67	19.55	22.42	14.49	10.84	**8.75**
	20–30%	27.49	28.12	26.93	27.69	18.98	**16.76**	17.19	27.03	28.85	26.23	31.54	24.46	19.22	**15.85**
	30–40%	45.36	41.68	39.75	41.24	31.91	29.80	**27.72**	42.51	40.24	36.98	45.10	37.66	31.28	**26.97**
L2^−^(10^−3^)	0–10%	1.23	0.96	0.84	0.64	0.54	0.46	**0.41**	0.82	1.14	0.73	0.97	0.72	0.52	**0.39**
	10–20%	3.93	2.55	2.15	**1.04**	1.49	1.58	1.46	3.08	3.03	1.92	3.12	2.81	1.78	**1.46**
	20–30%	6.92	4.87	4.00	**1.61**	3.07	3.21	2.95	6.00	5.56	3.42	5.79	5.23	3.56	**2.75**
	30–40%	12.99	9.21	8.01	**2.08**	6.38	6.66	5.98	10.60	9.23	6.35	9.77	8.91	6.53	**5.44**
FID^−^	0–10%	1.09	1.63	1.56	1.48	0.53	0.49	**0.43**	7.52	9.14	6.72	8.39	6.21	4.78	**3.83**
	10–20%	3.28	3.06	2.84	2.38	1.47	1.49	**1.36**	15.44	14.03	12.09	13.32	15.79	12.45	**11.79**
	20–30%	8.02	4.15	4.02	3.31	2.46	3.48	**2.45**	27.66	23.45	20.11	24.53	27.41	21.56	**19.81**
	30–40%	12.44	6.23	5.86	4.63	4.13	6.20	**4.01**	38.53	36.23	**28.43**	37.37	38.85	30.45	30.53

**Table 3:** Quantitative results of ablation studies on discontinuous missing regions, where + indicates the higher is better, while—indicates the lower is better, the **bold** indicates the best performance.

		CelebA-HQ									Paris Street View								
	MASK	L2	L1	CL	SL	IM	IM+AD (BL)	BL+AT	BL(Rcdc) +DFB	All Model	L2	L1	CL	SL	IM	IM+AD (BL)	BL+AT	BL(Rcdc) +DFB	All Model
PSNR ^+^	0–10%	39.40	41.49	41.47	41.45	41.46	41.60	41.55	41.61	**42.25**	40.49	41.63	41.31	41.18	41.69	41.74	42.14	42.01	**42.19**
	10–20%	32.87	34.76	34.13	34.05	34.75	34.88	34.85	34.93	**34.95**	33.66	34.57	34.23	34.01	34.63	34.70	35.00	34.90	**35.11**
	20–30%	29.35	30.95	30.26	30.19	30.95	31.09	31.04	**31.18**	**31.18**	29.92	30.57	30.20	30.03	30.54	30.69	31.03	30.99	**31.12**
	30–40%	28.35	30.02	29.26	29.18	30.02	30.14	30.12	**30.22**	30.18	28.82	29.51	29.16	28.96	29.63	30.03	29.92	29.87	**30.04**
SSIM ^+^	0–10%	0.983	0.990	0.990	0.990	0.989	0.989	0.989	0.989	**0.991**	0.987	0.989	0.989	0.989	0.989	0.989	**0.990**	**0.990**	**0.990**
	10–20%	0.947	0.966	0.962	0.962	0.965	0.965	0.965	0.965	**0.966**	0.954	0.962	0.960	0.960	0.961	0.962	0.964	0.963	**0.965**
	20–30%	0.907	0.935	0.925	0.925	0.933	0.933	0.933	0.934	**0.935**	0.909	0.922	0.916	0.916	0.920	0.921	0.925	0.923	**0.927**
	30–40%	0.872	0.913	0.898	0.899	0.911	0.912	0.912	0.912	**0.913**	0.878	0.896	0.889	0.889	0.896	0.897	0.902	0.899	**0.904**
L1^−^(10^−3^)	0–10%	4.54	3.80	1.73	1.70	3.77	3.76	3.78	3.76	**1.56**	2.11	1.73	2.59	2.54	1.74	1.73	1.64	1.64	**1.61**
	10–20%	8.15	7.00	6.03	5.92	6.91	6.85	6.92	6.86	**5.37**	7.03	5.68	6.59	6.51	5.78	5.67	5.36	5.35	**5.24**
	20–30%	14.70	11.16	11.12	10.80	10.96	10.80	10.92	10.76	**9.63**	13.75	11.69	12.39	12.22	11.56	11.18	10.60	10.59	**10.34**
	30–40%	19.32	13.75	14.61	14.25	13.50	13.32	**12.73**	13.29	13.67	18.06	14.67	15.87	15.68	14.54	14.54	13.78	13.76	**13.34**
L2^−^(10^−3^)	0–10%	0.19	0.14	0.13	0.14	0.14	0.14	0.14	0.13	**0.11**	0.16	0.14	0.16	0.16	0.14	**0.13**	**0.13**	**0.13**	**0.13**
	10–20%	0.64	0.45	0.51	0.52	0.45	0.44	0.44	0.43	**0.42**	0.60	0.53	0.58	0.60	0.52	0.52	**0.49**	0.50	**0.49**
	20–30%	1.39	1.02	1.19	1.20	1.01	0.99	1.00	0.97	**0.96**	1.42	1.31	1.44	1.49	1.29	1.28	1.23	1.23	**1.18**
	30–40%	1.72	1.23	1.46	1.48	1.22	1.20	1.20	1.18	**1.17**	1.81	1.63	1.79	1.87	1.65	1.61	1.54	1.51	**1.49**
FID^−^	0–10%	0.51	0.30	0.27	0.24	0.24	0.22	0.22	0.21	**0.18**	2.39	2.01	1.96	1.80	1.39	1.37	1.26	1.32	**1.13**
	10–20%	1.84	1.11	1.07	0.93	0.84	0.77	0.78	0.74	**0.70**	8.68	7.47	7.31	6.67	5.47	4.91	4.68	4.85	**4.58**
	20–30%	3.53	2.28	2.19	1.79	1.67	1.51	1.53	1.45	**1.38**	20.77	18.59	16.22	14.37	12.17	10.23	9.98	**9.76**	9.90
	30–40%	4.81	3.02	3.01	2.39	2.21	1.93	1.95	1.87	**1.82**	28.51	25.04	27.71	19.62	15.82	13.40	13.97	13.20	**12.98**

**Table 4:** Quantitative results of ablation studies on continuous missing regions, where + indicates the higher is better, while—indicates the lower is better, the **bold** indicates the best performance.

		CelebA-HQ									Paris Street View								
	MASK	L2	L1	CL	SL	IM	IM+AD (BL)	BL+AT	BL(Rcdc) +DFB	All Model	L2	L1	CL	SL	IM	IM+AD (BL)	BL+AT	BL(Rcdc) +DFB	All Model
PSNR ^+^	0–10%	34.70	37.65	37.00	37.03	37.17	37.29	37.44	38.19	38.30	34.39	36.92	35.49	35.67	37.16	37.26	37.47	37.58	**37.69**
	10–20%	27.95	29.81	29.08	28.98	29.72	29.84	29.93	29.95	**30.03**	28.22	29.94	28.90	28.95	30.13	30.22	30.47	30.32	**30.58**
	20–30%	24.77	26.39	25.71	25.58	26.31	26.44	26.44	26.53	**26.61**	25.31	27.01	25.79	25.83	27.04	27.11	27.28	27.38	**27.50**
	30–40%	21.65	23.14	22.54	22.42	23.02	23.15	23.30	23.34	**23.39**	22.81	23.93	22.95	22.83	24.01	24.19	24.23	**24.33**	24.31
SSIM ^+^	0–10%	0.975	0.982	0.980	0.980	0.980	0.981	0.981	**0.983**	0.982	0.974	0.979	0.976	0.976	0.979	0.980	0.980	0.980	**0.981**
	10–20%	0.931	0.947	0.938	0.939	0.944	0.945	0.946	**0.947**	**0.947**	0.924	0.935	0.926	0.925	0.935	0.936	0.937	0.937	**0.940**
	20–30%	0.881	0.904	0.887	0.889	0.900	0.901	0.901	**0.903**	**0.903**	0.868	0.886	0.869	0.868	0.886	0.887	0.889	0.889	**0.891**
	30–40%	0.821	0.851	0.824	0.824	0.846	0.846	**0.850**	**0.850**	0.849	0.811	0.830	0.803	0.800	0.830	0.832	0.833	0.834	**0.836**
L1^−^(10^−3^)	0–10%	6.62	2.86	3.13	3.04	5.15	5.09	5.03	2.69	**2.65**	4.43	3.96	4.33	4.20	3.71	2.97	2.95	2.92	**2.83**
	10–20%	14.89	8.85	9.69	9.42	10.80	10.61	10.38	8.39	**8.26**	13.37	10.44	11.53	11.22	9..91	9.13	8.92	9.04	**8.75**
	20–30%	23.84	16.61	18.18	17.76	18.22	17.88	17.60	17.50	17.19	23.63	17.88	20.30	19.97	17.37	16.68	16.82	16.93	**15.85**
	30–40%	42.68	29.01	31.61	30.93	30.57	30.24	28.71	**27.72**	**27.72**	37.10	29.85	33.05	32.72	28.58	27.81	27.57	27.36	**26.97**
L2^−^(10^−3^)	0–10%	0.66	0.43	0.52	0.52	0.46	0.45	0.45	0.43	**0.41**	0.60	0.45	0.53	0.55	0.43	0.42	0.42	0.40	**0.39**
	10–20%	2.12	1.51	1.77	1.77	1.55	1.52	1.52	1.47	**1.46**	2.08	1.58	1.86	1.94	1.53	1.52	**1.45**	1.51	1.46
	20–30%	4.14	3.08	3.58	3.62	3.15	3.06	3.01	2.96	**2.95**	3.78	2.85	3.60	3.76	2.86	2.91	2.81	2.81	**2.75**
	30–40%	8.32	6.34	7.24	7.30	6.50	6.35	6.08	6.00	**5.98**	6.61	5.67	6.92	7.18	5.53	5.42	5.51	**5.38**	5.44
FID^−^	0–10%	0.91	0.61	0.58	0.52	0.53	0.49	0.47	0.45	**0.43**	7.58	5.93	5.32	4.95	4.72	3.88	**3.59**	3.79	3.83
	10–20%	2.99	1.96	1.89	1.61	1.68	1.51	1.46	1.45	**1.36**	19.02	15.08	16.61	13.73	14.01	12.67	12.31	12.31	**11.79**
	20–30%	6.18	3.69	3.51	2.84	3.02	2.67	2.63	2.63	**2.45**	30.95	28.48	28.86	23.89	25.29	21.49	20.55	20.21	**19.81**
	30–40%	11.15	6.53	5.98	4.60	4.98	4.48	4.10	4.33	**4.01**	44.30	40.31	40.80	32.89	37.71	34.80	32.60	32.61	30.53

## References

[1] Li Z., He H., Tai H.-M., Yin Z., Chen F. (2014). Color-Direction Patch-Sparsity-Based Image Inpainting Using Multidirection Features. IEEE Trans. Image Process..

[2] Li Z., Liu J., Cheng J. (2019). Exploiting Multi-Direction Features in MRF-Based Image Inpainting Approaches. IEEE Access.

[3] Cao J., Zhang Z., Zhao A., Cui H., Zhang Q. (2020). Ancient mural restoration based on a modified generative adversarial network. Herit. Sci..

[4] Liu Q., Li S., Xiao J., Zhang M. (2019). Multi-filters guided low-rank tensor coding for image inpainting. Signal Process. Image Commun..

[5] Biradar R.L., Kohir V.V. (2013). A novel image inpainting technique based on median diffusion. Sadhana.

[6] Bertalmio M., Vese L., Sapiro G., Osher S. (2003). Simultaneous structure and texture image inpainting. IEEE Trans. Image Process..

[7] Yeh R.A., Chen C., Lim T.Y., Schwing A.G., Hasegawa-Johnson M., Do M.N. Semantic Image Inpainting with Deep Generative Models. Proceedings of the 2017 IEEE Conference on Computer Vision and Pattern Recognition (CVPR).

[8] Li X., Hu G., Zhu J., Zuo W., Wang M., Zhang L. (2020). Learning Symmetry Consistent Deep CNNs for Face Completion. IEEE Trans. Image Process..

[9] Chen M., Liu Z., Ye L., Wang Y. (2020). Attentional coarse-and-fine generative adversarial networks for image inpainting. Neurocomputing.

[10] Pathak D., Krahenbuhl P., Donahue J., Darrell T., Efros A.A. Context Encoders: Feature Learning by Inpainting. Proceedings of the 2016 IEEE Conference on Computer Vision and Pattern Recognition (CVPR).

[11] Yu J., Lin Z., Yang J., Shen X., Lu X., Huang T.S. Generative Image Inpainting with Contextual Attention. Proceedings of the 2018 IEEE/CVF Conference on Computer Vision and Pattern Recognition.

[12] Iizuka S., Simo-Serra E., Ishikawa H. (2017). Globally and locally consistent image completion. ACM Trans. Graph..

[13] Liu G., Reda F.A., Shih K.J., Wang T.-C., Tao A., Catanzaro B. Image Inpainting for Irregular Holes Using Partial Convolutions. Proceedings of the Medical Image Computing and Computer-Assisted Intervention—MICCAI’99.

[14] Yu J., Lin Z., Yang J., Shen X., Lu X., Huang T. Free-Form Image Inpainting with Gated Convolution. Proceedings of the 2019 IEEE/CVF International Conference on Computer Vision (ICCV).

[15] Ma Y., Liu X., Bai S., Wang L., Liu A., Tao D., Hancock E. (2019). Region-wise Generative Adversarial Image Inpainting for Large Missing Areas. arXiv.

[16] Sagong M.-C., Shin Y.-G., Kim S.-W., Park S., Ko S.-J. PEPSI: Fast Image Inpainting with Parallel Decoding Network. Proceedings of the 2019 IEEE/CVF Conference on Computer Vision and Pattern Recognition (CVPR).

[17] Qiu J., Gao Y., Shen M. (2021). Semantic-SCA: Semantic Structure Image Inpainting with the Spatial-Channel Attention. IEEE Access.

[18] Wang X., Girshick R., Gupta A., He K. Non-local Neural Networks. Proceedings of the 2018 IEEE/CVF Conference on Computer Vision and Pattern Recognition.

[19] Uddin S.M.N., Jung Y.J., Nadim U.S.M. (2020). Global and Local Attention-Based Free-Form Image Inpainting. Sensors.

[20] Yang J., Qi Z., Shi Y. (2020). Learning to Incorporate Structure Knowledge for Image Inpainting. arXiv.

[21] Liu D., Wen B.H., Fan Y.C., Loy C.C., Huang T.S. Non-Local Recurrent Network for Image Restoration. Proceedings of the Advances in Neural Information Processing Systems.

[22] Sun T., Fang W., Chen W., Yao Y., Bi F., Wu B. (2019). High-Resolution Image Inpainting Based on Multi-Scale Neural Network. Electronics.

[23] Yang C., Lu X., Lin Z., Shechtman E., Wang O., Li H. High-Resolution Image Inpainting Using Multi-scale Neural Patch Synthesis. Proceedings of the 2017 IEEE Conference on Computer Vision and Pattern Recognition (CVPR).

[24] Wang Y., Tao X., Qi X.J., Shen X.Y., Jia J.Y. Image Inpainting via Generative Multi-column Convolutional Neural Networks. Proceedings of the Advances in Neural Information Processing Systems.

[25] Wang Q., Fan H., Sun G., Cong Y., Tang Y. (2019). Laplacian pyramid adversarial network for face completion. Pattern Recognit..

[26] Mo J., Zhou Y. (2020). The image inpainting algorithm used on multi-scale generative adversarial networks and neighbourhood. Automatika.

[27] Hui Z., Li J., Wang X., Gao X. (2020). Image Fine-grained Inpainting. arXiv.

[28] Miyato T., Kataoka T., Koyama M., Yoshida Y. (2018). Spectral Normalization for Generative Adversarial Networks. arXiv.

[29] Isola P., Zhu J.-Y., Zhou T., Efros A.A. Image-to-Image Translation with Conditional Adversarial Networks. Proceedings of the IEEE Conference on Computer Vision and Pattern Recognition.

[30] Li C., Wand M. Combining Markov Random Fields and Convolutional Neural Networks for Image Synthesis. Proceedings of the 2016 IEEE Conference on Computer Vision and Pattern Recognition (CVPR).

[31] Vo H.V., Duong N.Q.K., Pérez P. Structural inpainting. Proceedings of the 2018 ACM Multimedia Conference (Mm′18).

[32] Nazeri K., Ng E., Joseph T., Qureshi F.Z., Ebrahimi M. (2019). EdgeConnect: Generative Image Inpainting with Adversarial Edge Learning. arXiv.

[33] Zheng C., Cham T.-J., Cai J. Pluralistic Image Completion. Proceedings of the 2019 IEEE/CVF Conference on Computer Vision and Pattern Recognition (CVPR).

[34] Heusel M., Ramsauer H., Unterthiner T., Nessler B., Hochreiter S. (2017). GANs Trained by a Two Time-Scale Update Rule Converge to a Local Nash Equilibrium. arXiv.

[35] Singh V.K., Abdel-Nasser M., Pandey N., Puig D. (2021). LungINFseg: Segmenting COVID-19 Infected Regions in Lung CT Images Based on a Receptive-Field-Aware Deep Learning Framework. Diagnostics.

